# Antimicrobial Nanoparticles Incorporated in Edible Coatings and Films for the Preservation of Fruits and Vegetables

**DOI:** 10.3390/molecules24091695

**Published:** 2019-04-30

**Authors:** Yage Xing, Wenxiu Li, Qin Wang, Xuanlin Li, Qinglian Xu, Xunlian Guo, Xiufang Bi, Xiaocui Liu, Yuru Shui, Hongbin Lin, Hua Yang

**Affiliations:** 1 Key Laboratory of Grain and Oil Processing and Food Safety of Sichuan Province, College of Food and Bioengineering, Xihua University, Chengdu 610039, China; xingyage1@163.com (Y.X.); 18408248463@163.com (W.L.); lxl0519@126.com (X.L.); xuqinglian01@163.com (Q.X.); gxl412326@163.com (X.G.); bxf1221@163.com (X.B.); xiaocuiliu777@126.com (X.L.); 13648022884@163.com (Y.S.); hongbin-ok@163.com (H.L.); yang1hua1@yeah.net (H.Y.); 2 Department of Nutrition and Food Science, Maryland University, College Park, MD 20742, USA; 3 Key Laboratory of Food Non-Thermal Processing, Engineering Technology Research Center of Food Non-Thermal Processing, Yibin Xihua University Research Institute, Yibin 644004, China

**Keywords:** chitosan coating, antimicrobial activities, physical characteristics, nanoparticle application, food preservation

## Abstract

Edible coatings and films (ECF) are employed as matrixes for incorporating antimicrobial nanoparticles (NPs), and then they are applied on the fruits and vegetables to prolong shelf life and enhance storage quality. This paper provides a comprehensive review on the preparation, antimicrobial properties and mechanisms, surface and physical qualities of ECF containing antimicrobial NPs, and its efficient application to vegetables and fruits as well. Following an introduction on the properties of the main edible coating materials, the preparation technologies of ECF with NPs are summarized. The antimicrobial activity of ECF with NPs against the tested microorganism was observed by many researchers. This might be mainly due to the electrostatic interaction between the cationic polymer or free metal ions and the charged cell membrane, the photocatalytic reaction of NPs, the detachment of free metal ion, and partly due to the antimicrobial activity of edible materials. Moreover, their physical, mechanical and releasing properties are discussed in detail, which might be influenced by the concentration of NPs. The preservation potential on the quality of fruits and vegetables indicates that various ECF with NPs might be used as the ideal materials for food application. Following the introduction on these characteristics, an attempt is made to predict future trends in this field.

## 1. Introduction

High decay rates pose a significant challenge to the storage of fruits and vegetables leading to nutrient loss and the spread of microorganisms responsible for degradation [1], leading to huge fresh produce losses due to inappropriate storage conditions all over the world every year. Therefore, innovative technologies such as edible coatings and films (ECF) and controlled atmosphere packaging are suggested as possible solutions for maintaining the quality of agricultural products during storage and shelf time [2,3,4]. The spread of harmful microorganisms poses a severe threat to fruit quality and human health [5], encouraging extensive research into the combined application of nano-biocomposites and edible coatings [6,7].

Many studies suggested possible environmentally-friendly alternatives as ECF matrixes, including but not limited to carbohydrate-based polymers such as chitosan (CS), starch and β-cyclo-dextrin (β-CD) [4,6,7,8]. For examples, chitosan can form a thin film on the surfaces of pulps, which might control the decay and keep the quality of fruits by inhibiting the growth of bacteria and fungi, and reducing their respiration rates [4]. Moreover, starch has been considered as another suitable coating material due to its properties of wide availability, renewable and biodegradable biopolymer with low cost [6,8]. However, various challenges were proposed, such as weak barrier qualities, low mechanical characteristics, together with low antimicrobial activity [9], which prompted the development and application of edible coatings containing inorganic nanofillers [10].

The preparation and application of bio-nanocomposite packaging with polymeric materials and inorganic nanoparticles (NPs) for food preservation are expected to be developed in the future [11,12,13]. Of the various types of inorganic NPs, titanium dioxide (TiO_2_) are always used as an antimicrobial agent to coat different materials for further application in many food products [13]. The photocatalytic behavior of TiO_2_ is hugely relied on visible light irradiation, as well as UV light, which is responsible for activating its antimicrobial properties [14,15,16]. Meanwhile, the electrocatalytic activity of silver nanoparticles (AgNPs) could provide the excellent antimicrobial property for its extensive application by incorporating into edible polymers as an active food packaging [16,17]. ZnO, in conjunction with antimicrobial activity can act as a permeation barrier for further application [10,11]. Furthermore, it was found by other researchers that various sizes of NPs had influence on the mechanical and physical properties, filtering ultraviolet (UV) light, as well as their antimicrobial activity [11,12].

Some interesting works on preparation and property characterization of ECF with NPs have been conducted by researchers [3,14]. As reported by Cano et al. [14], TiO_2_-based NPs were incorporated into the CS-based matrix to obtain different nano-biocomposites. Moreover, Xu et al. [5] found that the composite coating with graphene oxide (GO), CS and TiO_2_ NPs at the ratio 1:20:4 exhibited excellent antibacterial activities against *Aspergillus niger* and *Bacillus subtilis*, which might induce the cell membrane rupture. Andrade et al. [18] also showed that the β-CD-coated-Ag NPs reduced more than 99% *E. coli* and *Pseudomonas aeruginosa* CFU compared to the control samples without silver addition. Shankar et al. [19] indicated the incorporation of sulfur nanoparticles (SNP) could enhance the hydrophobicity, mechanical strength, and water vapor barrier property as well as antimicrobial activity of chitosan film. Furthermore, SNP capped with chitosan film exhibited the highest antimicrobial activity against *E. coli* and *Listeria monocytogenes* with complete sterilization within 6 h and 12 h, respectively.

Since not many studies are currently available involving the influence of ECF with NPs on the quality of fresh produce in storage. Yu et al. [20] examined harvested jujube to determine the combined effect of CS and nano-silicon on its quality characteristics. The quality indexes including decay incidence, weight loss, the red index, and respiration rate, exceeded those of the control samples after storage for 32 d at an ambient temperature. As reported by Shi et al. [21], by reducing the browning index, as well as obstructing polyphenol activity and the increase of malondialdehyde (MDA), the film of CS/nano-silica significantly extended the longevity of fresh longan. They indicated that the CS/nano-silica coating showed promise for the quality preservation of fresh longan during prolonged storage.

In recent years, the preparation and application of ECF and nanostructured materials are increasing for extending the shelf life of fresh agricultural products. These research works have also been summarized by researchers. The different types of bio-based materials, their applications as packaging materials, and future trends were reviewed by Sorrentino et al. [22]. Moreover, Dutta et al. [23] and Xing et al. [4] have introduced the different prepared technologies, the antimicrobial activity and mechanism of chitosan based films. Falguera et al. [24] also concluded the latest developments on the structures, active properties, and the application trends of edible coatings and films. Furthermore, Carbone et al. [25] have reported the advanced technology by using AgNPs-doped edible polymers and oils to provide the preservation for food products. The applications of nanotechnology in the food industries had been concluded by Bajpai et al. [26]. Moreover, the nanocomposite packaging materials from the views of properties, food applications and safety had also been reviewed by Sothornvit [27]. Although several edible-based coating and nanostructured materials were developed and applied in food packaging systems, until now, no detailed report has been published to summarize the preparation and various properties of ECF with antimicrobial NPs for the storage of vegetables and fruits.

Therefore, the purpose of this paper is to review the preparation and various properties of ECF with NPs for the storage of fresh produce. The first part was to conclude the properties of several main coating materials and to introduce the preparation techniques of ECF with NPs reported by many researchers. In the next, the antimicrobial activities and mechanisms, physical and mechanical characterization of ECF with NPs were summarized. In addition, thermal stability, structural characteristics, and the properties including gas modification, induction defense and ion release of ECF with NPs were reviewed. Finally, its applications in the storage of fruits and vegetables were introduced and the useful insights for its further research were provided.

## 2. Preparation Technologies of ECF with Different NPs

### 2.1. Structure, Composition and Properties of the Main Edible Coating Materials

**Figure 1:**
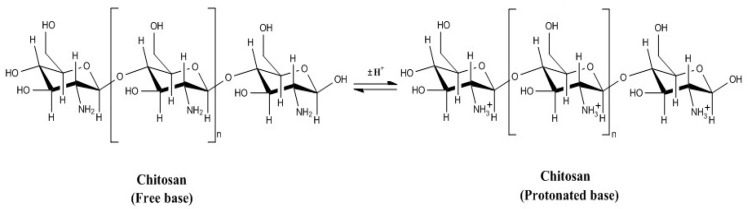
The acid-base equilibrium attributes of CS.

Chitosan with different functional groups can be used as a natural carbohydrate biopolymer for keeping fresh of fruits and vegetables [1,4]. The acid-based equilibrium features of CS is illustrated in Figure 1, which denote a linear polysaccharide consisting of (1,4)-linked 2-amino-deoxy-β-d-glucan with one primary amino group, as well as two free hydroxyl groups [4,28,29,30,31]. While dissolving in diluted acidic solutions [1,4,28], positively charged amino groups can successfully react with numerous negatively charged surfaces of polymer materials and cells [23]. Due to its exceptional biocompatibility and film-forming properties, CS is widely applied as an edible coating film during the storage of fresh produce, and was deemed as generally recognized as safe (GRAS) in 2005 by the food and drug administration (FDA) according to the scientific procedures for use in food (FDA/CFSAN) [1,4].

Starch is a natural polymer with the properties of cheap, biodegradable, renewable and plentiful, and shows promise as a coating material [32,33]. It consists of amylose, amylopectin, as well as two macromolecules [34]. As a linear polymer, amylose consists of glucose units connected with α-1, 4-bonds, while short linear chains branched onto longer chains with α-1, 6 linkages denote the extensively branched polymer amylopectin [34,35]. Despite starch films being devoid of color, taste, and flavor, as well as being translucent or transparent, they are defined by two primary challenges namely their high sensitivity to moisture and low mechanical characteristics [32,34,35,36,37].

Shellac is a natural resin generated from insects, which is frequently employed by the food industry to glaze and treat the surfaces of citrus fruits and confectionary products to avoid damage during storage [15,38]. It exhibits a chemical structure with a large number of carbonyl and carboxyl groups [38,39]. Although shellac exhibits no antimicrobial effect, it is always used in various food applications as the non-toxic binder and the coating material [15]. More importantly, shellac being approved by the FDA as GRAS can be utilized for indirect food contact. Furthermore, shellac films display exceptional adherence capacity to various surfaces with excellent rigidity, strength and high gloss [15,38].

**Figure 2:**
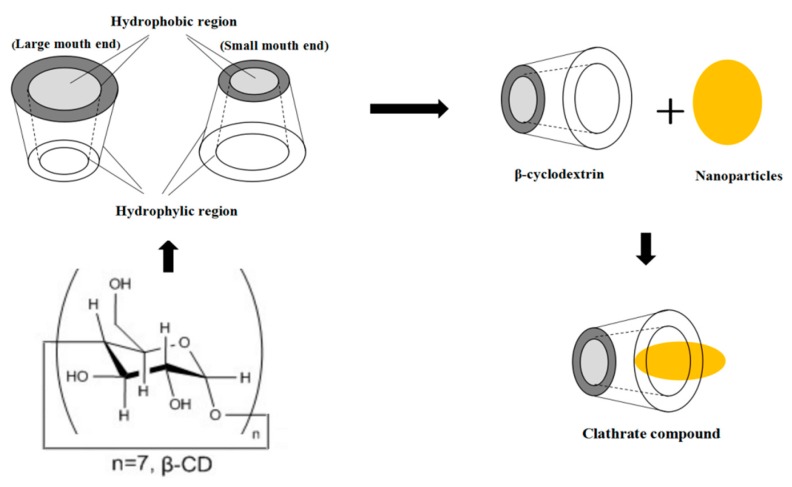
The structure of β-CD and the simulated enveloped process of β-CD with NPs.

Polycyclic glucose oligosaccharides known as cyclodextrins (CDs) are composed of 6, 7 or 8 glucopyranose units connected with β-1,4-glucosidic bonds referred to as α-, β-, or γ- cyclodextrin, respectively [18]. The structure in Figure 2 illustrates β-CD molecules consisting of a hydrophilic exterior with hydroxyl groups, as well as a hydrophobic central cavity. By forming inclusion complexes that are water soluble, low polarity and non-polar molecules displaying suitable shape and size are allowed to solubilize [18,40]. However, the cavity of β-CD is too small for encapsulating metal NPs. The simulated enveloped process of β-CD with NPs is shown in Figure 2 and β-CD can be used as the nanoparticle stabilizer and can simply bind to the NPs via chemisorption through hydroxyl groups [41].

Composed of a linear series of sulfated galactans, carrageenan is a hydrocolloid extracted from red algae. It is a water-soluble, natural product with the potential to be employed as a film-forming substance [42]. The number and location of a sulfated ester on 3,6-anhydro-d-galactose residues determine the specific classification of the galactans. Similar to other hydrocolloid-based films, they display inadequate water-vapor-barrier properties [42,43].

Kefiran is a naturally occurring polysaccharide with characteristics that include gelatinization, film-formation and texturization. These properties are adequate for its utilization in food packaging [44,45,46,47]. Consisting of equal parts of d-galactose and d-glucose, kefiran is a water-soluble substance derived from glucogalactone and is responsible for enhancing milk gel viscosity, as well as its viscoelastic qualities [44,45]. However, insufficient mechanical strength and high moisture permeability denote some of the unique challenges of films produced from kefiran [44].

Consisting of β-d-glucose, linear carboxymethyl cellulose (CMC) is an essential, water-soluble cellulose derivative [47,48], which is cost-efficient, non-allergenic, transparent, non-toxic, easy to process and possesses preferable film-forming qualities [48,49,50]. CMC is exceptionally susceptible to water vapor even though it displays sufficient capability to form adequate transparent films and is an effective inhibitor of oxygen and lipids [50].

A galacturonan backbone with homogalacturonans (-1,4-galacturonan) and rhamno-galacturonans branched by -1,4-galactan and -1,3- or -1,5-arabinan chains form the pectin-like framework of the soluble soybean polysaccharide (SSPS), which is derived from the cell-wall substances of soybean cotyledons [51,52]. While SPSS is commonly used for producing edible films, which are water-soluble and colorless, some pertinent challenges include mechanical vulnerability, high gas patency, as well as elevated absorption capacity for water moisture [53,54,55].

With cost efficiency and versatility among its attributes, polylactic acid (PLA) presents a new kind of biodegradable substance, which is manufactured from sustainable plant materials such as corn starch and sugar beets [56,57], and obtained FDA certification approving food-contact utilization [58]. However, no antimicrobial effect is evident in the pure PLA film [59]. As a natural, linear and aliphatic polyester, PLA films exhibit a high level of transparency and extremely water resistant [59,60,61]. They are frequently employed to enhance food preservation and prolong the storage life of fresh produce [57,59,60,61].

As a popular substance obtained from the byproducts of corn-refining, zein is produced from corn gluten meal, and the films derived from it exhibit useful qualities such as biodegradable, transparent, as well as acting as an oxygen barrier [62,63,64]. Zein protein (ZP) forms part of the protein bodies and is located in corn endosperm, and three specific fractions namely α, β and γ zeins were determined and isolated according to disparate levels of solubility in aqueous alcohol mixtures [65].

Whey protein as a by-product of cheese is a yellow-green liquid and well-known material to form biodegradable films [44,66]. The protein content of whey protein concentrates (WPC) ranged between 35-90%, while that of whey protein isolates (WPI) exceeds 90%. Both of them can be used to produce biodegradable films that are transparent, flexible and devoid of flavor and color [66,67]. Whey protein film exhibits a fairly high moisture permeability, as well as unique mechanical characteristics while inhibiting oxygen penetration [44,66].

### 2.2. Preparation of ECF with NPs

**Figure 3:**
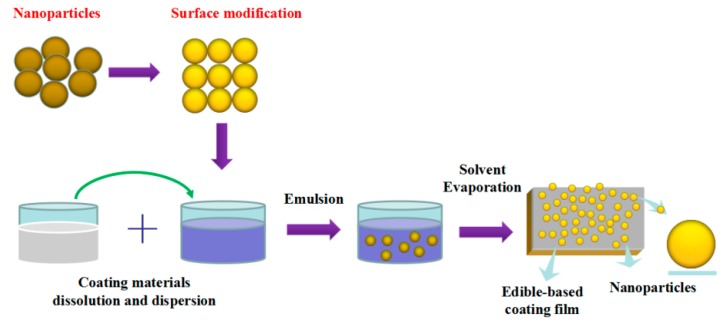
The general technology to prepare the ECF with NPs.

Many kinds of edible coatings with and without antimicrobial agents such as essential oils have been developed and investigated [1,4,68,69,70]. In recently years, the reported works on NPs as antimicrobial agents and the preparation ECF with different NPs are increasing [4,71,72,73]. In this section, the different NPs and main edible-based materials used in coating films are summarized, which is listed in Table 1 and introduced as follows. However, the uniform dispersion of NPs is a critical problem in the solution system. As shown in Figure 3, the general technologies to prepare the ECF with NPs were summarized and reported, which includes several steps such as the surface modification of nanoparticles, coating materials dissolution, emulsion formation, and solvent evaporation. These preparations were conducted in order to solve the problem of NPs agglomeration and develop these complex coatings/films with high antimicrobial activity for application.

#### 2.2.1. Preparation of Edible Coating Films with TiO_2_NPs

Yemmireddy and Hung [15] had prepared total six different suspensions by mixing TiO_2_ NPs with shellac, polyurethane, and polycrylic in a porcelain mortar for about 15 min. The obtained suspensions were further treated in an ultrasonic water bath for 1 h in order to avoid the aggregation of TiO_2_ NPs.

Zhang et al. [73] developed a CS/WPI film incorporated with modified TiO_2_ NPs. Firstly, CS, sodium laurate-modified TiO_2_ NPs and glycerin were added into an acetic acid solution and stirred for 2 h. Then, WPI was dispersed in deionized water. These two solutions were mixed and the mixture was adjusted to pH 3. After being stirred and degassed under vacuum, the obtained solution was casted over the plastic Petri dish and dried at 60 °C in oven for 16 h. On the other hand, the TiO_2_/plasticized CS nanobiocomposites and the graphene oxide-chitosan (GO-CS)-TiO_2_ coating were prepared by Cano et al. [14] and Xu et al. [5] with the similar technology having a little modifications, respectively.

Oleyaei et al. [74] have prepared potato starch film with sodium montmorillonite (MMT) and TiO_2_. Firstly, starch was mixed with distilled water and glycerol at room temperature (25 °C) for 5 min. This suspension was treated at 90 °C for 30 min and agitated. Then, MMT and TiO_2_ were dispersed separately in distilled water by sonication for 60 min at room temperature and were added to the aqueous dispersion of starch. The mixture was continuously stirred for 10 min. After being vacuumed to remove air bubbles from solutions, the solution was poured into a polystyrene tray and dried at 60 °C for 15 h in an oven. Goudarzi et al. [8] and Goudarzi et al. [34] had also developed the starch-TiO_2_ NPs coating film.

Teymourpour et al. [51] had prepared SSPS biocomposites with nano TiO_2_. TiO_2_ were first dispersed in deionized water and homogenized for 15 min. The obtained solutions were used to prepare the aqueous SSPS dispersions. A mixture of glycerol and sorbitol was prepared and added. SSPS nanocomposites were treated at 85 °C for 1 h. After the solution cast on Preplex plates fitted, the films were dried at 25 °C and 50% relative humidity (RH). After being peeled, the dried films were stored at 25 ± 2 °C and 55 ± 5% RH. Furthermore, the TiO_2_/SSPS composite films were also prepared by Salarbashi et al. [54] using the similar technology.

Zolfi et al. [44] and Alizadeh-Sani et al. [66] had prepared the WPI-based coating films with TiO_2_ NPs. The preparation of WPI/cellulose nanofibers (CNFs) with TiO_2_ NPs was introduced by Alizadeh-Sani et al. [66] as follows. Firstly, TiO_2_ NPs were added to WPI/CNFs solutions with stirring and sonication. Then, rosemary essential oil (REO) was added to the composite film solution and homogenized for 10 min. WPI/CNFs suspension with TiO_2_ NPs and REO were cast onto a petri dish and then dried using vacuum oven at 30 ± 1 °C and 50 ± 6% RH for 24 h.

The CMC-well-dispersed ternary nanocomposite coating films containing sodium montmorillonite (Na-MMT)-TiO_2_ was developed by Achachlouei et al. [50]. Firstly, Nano-TiO_2_ powder and ternary nanocomposite films containing Na-MMT were dispersed in distilled water with stirring and sonicating, respectively. Then, CMC was added to distilled water (95 °C) and mixed for 1 h. The Na-MMT and TiO_2_ sonicated solutions were added into the CMC solution, respectively. After glycerol was combined and stirred for 15 min, the solutions were cooled at room temperature. The obtained solutions were cast onto a polystyrene petri-dish and dried at 50 °C for 30 h in an oven.

#### 2.2.2. Preparation of Edible Coating Films with AgNP

AgNPs conjugated with CS as the composite films were conducted by Mathew and Kuriakose [75], Lin et al. [76], Kumar-Krishnan et al. [77] and Davoodbasha et al. [17]. Here introduces the prepared technology reported by Lin et al. [76]. Firstly, EDC (1-ethyl-3-[3′-dimethylamino-propyl] carbodiimide)/NHS (N-hydroxysuccinimide) was added to AgNPs solution with a carboxylate and the solution was incubated at 25 °C for 4 h with shaking. Then, the CS/cellulose film was submerged into the solution for 24 h with shaking. During the preparation process, the carboxyl groups of AgNPs were activated by EDC and NHS and subsequently reacted with the amino groups of CS in the composite film. Finally, the modified film was taken out and washed with the deionized water.

Starch-polyvinyl alcohol (PVA)-AgNPs film was prepared by Cano et al. [78]. Firstly, starch was dispersed in an aqueous solution at 95 °C for 30 min with stirring. After homogenized, PVA was incorporated into the gelatinized starch dispersion and glycerol was added. Afterwards, starch-PVA film forming dispersions containing AgNPs were obtained by the reduction of silver nitrate using UV light. The films including PVA-GO-Ag-starch films had prepared by Usman et al. [79]. PVA was dissolved in DI water by heating at 90 °C for 1 h. GO suspension was made by adding GO flakes in DI water by ultra-sonication for 3~h. Both suspensions were mixed and stirred for 1~h. Then, starch and AgNO_3_ were added into the obtained solution and mixed. This solution was placed in an autoclave at 121 °C and 15 psi pressure for 1 h. Followed by cooling and casting onto glass petri dish were conducted and dried at room temperature for 3 d.

AgNP stabilized with β-CD (AgNP-β-CD) was developed by Andrade et al. [18]. The aqueous NaOH, aqueous glucose and aqueous β-CD solutions were mixed and heated. An aqueous AgNO_3_ solution was dropwise after the solution reached to 60 °C. After that, the reaction product was dialyzed with deionized water. Then, the stabilized AgNPs were lyophilized and AgNP-β-CD powder was obtained.

Orsuwan et al. [80] had developed the films of agar and banana powder composite with AgNPs. Firstly, film forming solution was prepared by dissolving agar and banana powder in distilled water and heating at 90 °C for 20 min with stirring. Then, after the glycerol added, the aqueous solution of AgNO_3_ was added into the agar and banana powder (A/B) film solution and heated at 90 °C for 4 h with stirring. The solutions were cast onto the leveled Teflon film coated glass plates and dried at room temperature for 48 h.

#### 2.2.3. Preparation of Edible Coating Films with ZnONP

Malini et al. [81] had reported the preparation of nanocomposite CS/ZnO membrane. CS was added and dissolved in an aqueous acetic acid solution. After silica particles added with stirring, the solution was poured onto a glass plate for drying. The dried membrane was immersed in an aqueous NaOH solution and kept for 2 h at 80 °C for dissolving the silica particles in order to generate a porous membrane. Afterwards, the porous membrane was washed with distilled water and then immersed in an aqueous glycerol solution for 30 min for removing the excess glycerol solution. Finally, film discs were cut and introduced in the suspension of nano ZnO in distilled water under stirring at 28 °C for 24 h. The CS/ZnO discs were separated, rinsed with distilled water and air dried.

Nafchi et al. [82] had prepared the sago starch films incorporated with nanorod-rich ZnO (ZnO-N). Firstly, ZnO-N was dispersed in water stirred for 1 h and sonicated for another 30 min. This solution was used to prepare the aqueous starch dispersion. A mixture of sorbitol and glycerol were added. Starch nanocomposites were treated at 85 ± 5 °C for 45 min. After starch gelatinization completed, the solution was cooled to room temperature. Finally, the dispersion was cast on the plates and dried at 25 °C and 50% RH in a humidity chamber.

Composite films of fish protein isolate (FPI) and fish skin gelatin (FSG) blend incorporated with basil leaf essential oil (BEO) and ZnO nanoparticle (ZnONP) were prepared by Arfat et al. [83]. Firstly, FPI was added with distilled water and homogenized for 1 min. Its pH was adjusted to 3. The obtained solution was filtered and adjusted the protein concentration. Then, glycerol was added as a plasticiser. The mixtures were stirred for 30 min at room temperature and used for preparing blend film-forming suspension (FFS). On the other hand, FSG powder was dissolved in distilled water. Then its pH was adjusted to 3 and then heated at 70 °C for 30 min. Afterward, glycerol was added and both FPI and FSG solutions were mixed. Moreover, ZnONP suspended previously in distilled water was added in this mixture. The obtained FPI/FSG/ZnONP suspension was stirred for 5 min and then homogenised for 30 s. Furthermore, BEO previously mixed with tween 20 was added to this above suspension. The film-forming suspension was obtained by homogenising and stirring.

Espitia et al. [84] developed nanocomposite films of methyl cellulose (MC) incorporated with pediocin and ZnONP. ZnONP were mixed with deionized water and then sodium pyrophosphate was added. The ZnONP dispersion was sonicated for 23 min. Then, glycerol was added to the nanoparticle solution, which was heated at 80 ± 2 °C to solubilize the methyl cellulose. Pediocin was added to this solution with MC. Moreover, the gelatin/ZnONP film and carrageenan/ZnONP (CZ) based films were prepared by Shankar et al. [19] and Meindrawana et al. [43] using the similar procedure, respectively.

Akbariazam et al. [52] has developed the SSPS-ZnO film. Firstly, ZnO was dispersed in water and homogenized for 15 min. This solution was used to prepare the SSPS dispersion. A mixture of sorbitol and glycerol was added to the formulation. Dispersions were heated to 75 ± 5 °C and stirred for 1 h. Then, the dispersions were cast on plates, dried at 25 °C and 50% RH in a humidity chamber. After peeled, the films stored in a closed desiccator containing saturated magnesium nitrate at 25 ± 2 °C and 50% ± 5% RH.

#### 2.2.4. Preparation of Edible Coating films with Other NPs

Yu et al. [20] have reported the preparation of CS-based coating with nano-silicon dioxide. Firstly, the mixed solution of CS, nano-silicon dioxide and deionized water was dispersed for 15 min by ultrasonication. Then, sucrose ester of fatty acid was added and dispersed for 5 min. The CS coating with nano-silicon dioxide was obtained after adding the glycerine and being dispersed. The CS solution with nano-silicon dioxide was prepared by Sun et al. [85]. Moreover, Shi et al. [21] and Song et al. [86] had introduced a preparation technology of the CS/nano-silica coating film.

Hasheminya et al. [47] had prepared the Kefiran-CMC films containing copper oxide nanoparticles (CuONPs). Firstly, the kefiran solution and CMC solution in distilled water were prepared, respectively. These two solutions were mixed and glycerol was added. Then, the stirring was conducted another 15 min. Subsequently, CuONPs were added to the kefiran-CMC solution and conducted sonication. Finally, the obtained solution was poured into glass plates and dried at 25 °C for 72 h.

Shankar et al. [19] had developed the SNP-CS films. SNP was added into acetic acid solution and dispersed with ultrasonication and homogenization. Then, glycerol was added and the mixture was stirred for 20 min. Afterwards, CS was added with continuous stirring for 20 min. Finally, the film suspension was heated with stirring at 80 °C for 30 min and was cast onto a glass plate. After being dried at room temperature for 2 days, the completely films were peeled and stored.

#### 2.2.5. Preparation of Edible Coating Films with Mixed NPs

Lin et al. [87] had developed a Ag-TiO_2_-CS nanocomposite as an antibacterial coating. After modification of CS, the CS adipate aqueous solution was prepared and mixed with AgNO_3_ solution. The mixture was stirred at 50 °C for 1 h. Meanwhile, TiO_2_ NPs were dispersed in distilled water with sonication. Subsequently, the TiO_2_ dispersion was added into the Ag/CS adipate solution. The solution was treated with UV radiation for 10 min. The resulting Ag was deposited on the surface of TiO_2_ NPs and a complex was formed with CS via Ag-NH_3_ coordination bonds through the amino groups on CS. On the other hand, Li et al. [88] and Zaharia et al. [89] used the similar technology to prepare the CS/Ag/ZnO blend coating films.

Chi et al. [57] developed the PLA/BEO/nanocomposite films. Briefly, PLA and BEO were dissolved in dichloromethane. Then, 2% nano-TiO_2_, and 1% nano-Ag based on PLA dry matter were added into the PLA/BEO dichloromethane solution and the solution was stirred for 10 h at room temperature. Finally, the solutions were poured onto the plate and dried at room temperature.

Arfat et al. [90] had prepared the FSG films containing Ag-Cu NPs nanocomposit. Ag-Cu NPs with selected loadings were mixed well in distilled water; the dark grey colored suspensions were stirred for 5 min and further homogenization for 1 min. Then, gelatin powder was added into the above prepared Ag-Cu NPs suspensions and glycerol was added to the suspension under stirring. The final volume was adjusted to 100 mL using distilled water and heated at 70 °C for 30 min. The suspensions were sonicated for 30 min and stirred for 2 h at room temperature. After being degassed for 10 min by sonication, the NPs nanocomposite solution was obtained.

Zein protein (ZP) nanocomposite films were prepared by Kadam et al. [64]. A film forming solution was prepared with ZP, aqueous ethanol, glycerol and PEG (polyethylene glycol)-600. The pH of the film-forming solution was then adjusted to 8.0. The solutions were heated to 62 ± 2 °C for 15 min under stirring and cooled to room temperature for 15 min. For ZP solutions with the core-and-shell NPs (TiO_2_ as core and SiO_2_ as shell), 1.5% *w*/*w* NPs were added to film-forming solution prior to heating. After subjected to ultrasonication, the ZP with NPs solutions were cast on dishes and dried at ambient conditions (24 ± 1 °C) for 48 h. Afterwards, they were stored in 50 ± 2% RH chamber for 24 h before peeling.

## 3. Antimicrobial Activity and Mechanism of ECF with NP

### 3.1. Antimicrobial Activity of ECF with NPs

**Figure 4:**
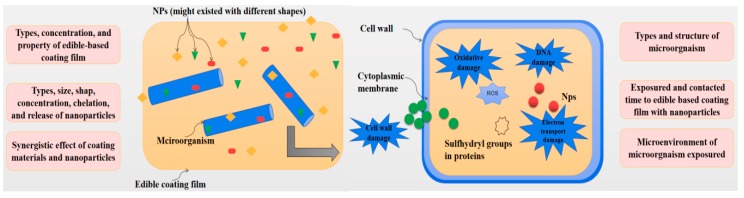
Factors affected the antimicrobial activity of ECF with NPs.

Since postharvest fresh produce is vulnerable to many microorganisms responsible for degradation, its quality can be considerably affected by leading to a shortened shelf-life. Edible coatings such as polysaccharide and protein-based coatings could be used to create thin films, which are applied on the surfaces of fruits and prevent bacteria growth, as well as the growth of yeast and molds [91,92]. The antimicrobial activities are affected by several factors. As illustrated in Figure 4, the efficacy in reducing microbial growth with these coatings might be primarily due to the type, concentration, and properties of coating films as well as the type, size, shape, concentration, chelation, release and photocatalytic properties of incorporated NPs [93,94,95,96,97,98]. Furthermore, the antimicrobial activity of edible coatings is influenced by the synergistic effect of coating materials and NPs in the barrier, the types and structure of tested microorganism, and the tested conditions (Figure 4) [4,12,95,96,97,98]. On the other hand, the antimicrobial activities of various ECF with NPs were also investigated by several researchers, which are summarized and listed in Table 2.

Alizadeh-Sani et al. [66] indicated that combining TiO_2_ and rosemary essential oil (REO) to form WPI/CNFs films resulted in higher antimicrobial activity against Gram-positive bacteria including *S. aureu* and *L. monocytogenes* compared to Gram-negative bacteria including *E. coli O*_157_:*H*_7_, *S. enteritidis* and *P. fluorescens*.

Huang et al. [99] stated that it was possible to increase the log reduction for *L. monocytogenes* to >4 log CFU using TiO_2_-polylactide composites illuminated with UV-A. This composite film illuminated with UV-A exhibited the equally antimicrobial activities against *Salmonella Typhimurium* and Shiga toxin producing *E. coli*. Moreover, Teymourpour et al. [51] and Salarbashi et al. [54] found that SSPS-TiO_2_ bio-NC films displayed an excellent antibacterial action against *S. aureus* and *E. coli*. Similarly, its antibacterial action against *S. aureus* was also observed by Salarbashi et al. [54]. Kumar-Krishnan et al. [77] demonstrated that the antibacterial potency of CS/AgNP composite films against *E. coli* and *S.aureus* was observed. Elevated levels of Ag successfully increased the antibacterial effect of both CS/AgNP and CS/Ag^+^ ion composite films. While morphology, particle size, colloidal stability, surface area, and purity were the determining factors for AgNPs activity (Figure 4) [100]. The results of Jia et al. [101] showed that the silver/CS Janus NPs exhibited a high bactericide effect against *Salmonella choleraesuis, S. aureus*, *B. subtilis*, and *E. coli* bacteria, as well as *Botrytis cinerea* fungi [19,79,80,87]. The results of a study conducted by El-Sherbiny et al. [102] indicated that elevated AgNPs concentrations and the in situ development of AgNPs on the surface of poly(ε-caprolactone)/curcumin/grape leaf extract-Ag hybrid NPs seemed compelling in its efficacy against bacteria including *E. coli*, *S. aureus*, *S. enterica*, *P. aeruginosa, B. subtilis,* and fungi including *Candida albicans* and *Aspergillus flavus*. Davoodbasha et al. [17] reported that the growth inhibition zone induced by Ch3Ag1 (3% CS solution mixed with 1 mM of AgNO_3_) was present for *E. coli*, *P. aeruginosa*, *Vibrio parahaemolyticus*, *Vibrio vulnificus*, *S. aureus,* and *B. cereus*. Furthermore, the antimicrobial properties of AgNPs-encapsulated chitosan against *S. aureus*, *E. coli*, *Aspergillus* *terreus* and *Aspergillus flavus* were also observed by Mathew and Kuriakose [75]. The growth inhibition zone caused by the Ch3Ag1 was 11 mm for the *Candida albicans*, but no inhibition was observed for the *Aspergillus parasiticus*. However, *A. parasiticus* pigmentation was repressed to some degree with elevated levels of the AgNP concentration. More importantly, the agglomeration of NPs became significant at higher concentrations, consequently exhibited a decline in contact between the cell walls of the bacteria and the specific particles [17,103]. Lin et al. [76] also indicated that both antimicrobial agents (CS and AgNPs) could act synergistically to enhance their antimicrobial efficacies [31]. Cano et al. [78] reported that Ag-loaded films exhibited antimicrobial activity against *A. niger*, *Penicillium expansum*, *Listeria innocua,* and *E. coli*, which appeared to be concentration dependent [75,104].

Malini et al. [81] determined that bacterial growth appeared to be inhibited by the CS/ZnO NC membrane, while Gram-positive *Bacillus substili* seemed to be less vulnerable to this process than Gram-negative *Klebsiella planticola* [105]. Al-Naamani et al. [106] found that following a 24 h incubation period, chitosan-ZnO nanocomposite showed an excellent inhibition of bacterial growth including *Salmonella enterica*, *E. coli,* and *S. aureus*. Furthermore, polyethylene films (PE) subjected to CS-ZnO NC coatings prevented the formation of food pathogens completely [43,52,83,107]. Carvalho et al. [13] indicated that a smaller the particle size encouraged an elevated concentration of nano or micro particles, therefore increasing the inhibiting efficiency. On the other hand, as Arfat et al. [83] reported that the FPI/FSG films incorporated with 100% BEO and ZnONP exhibited strong antibacterial activity against *L. monocytogenes* and *P. aeruginosa*.

According to Hasheminya et al. [47], the bacteria of *S. aureus* and *E. coli* were effectively restricted by the kefiran-CMC-CuONPs bio-NC. While Shankar et al. [108] indicated that *E. coli* and *L. monocytogenes* were significantly inhibited by CS capped SNP composite film, which displayed the highest antimicrobial activity with complete sterilization within 6 h and 12 h, respectively. Furthermore, Li et al. [88] indicated that bacteria such as *E. coli, B. subtilis, Aspergillus, Rhizopus, Penicillium, S. aureus,* and *yeast* responded exceptionally well to the antimicrobial effect of the CS/Ag/ZnO film combinations. More importantly, the chelating function among different NPs might also affect by the antimicrobials efficacy of separate NPs [109,110,111,112]. As indicated by Lungu et al. [111], AgNPs appeared to improve the antibacterial properties of both Ag-ZnO NPs without UV radiation. The higher antibacterial activity of Ag-ZnO NPs (chemical deposition (CD)) was found that that of ZnO NPs and Ag-ZnO NPs [111]. Erdural et al. [112] also reported that the highest photocatalytic antibacterial activity was obtained at over 92 wt.% SiO_2_-TiO_2_ surface. While Lin et al. [87] indicated that nano-Ag particles displayed lower antibacterial activity than the Ag/TiO_2_/CS NC at similar concentrations.

### 3.2. Antimicrobial Mechanism of ECF with NPs

**Figure 5:**
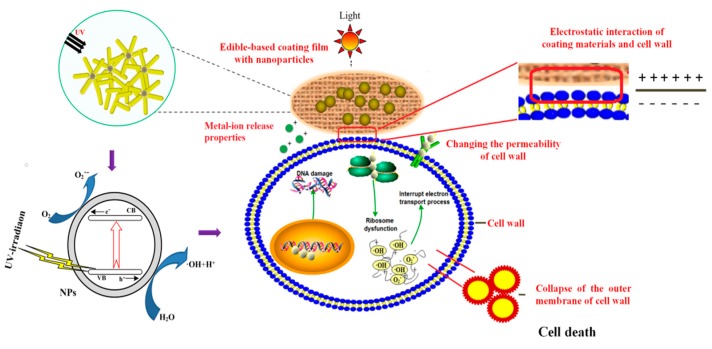
Proposed antimicrobial mechanism of ECF (such as chitosan-based coating) with NPs.

Several popular mechanisms, as shown in Figure 5, have been reported and concluded by researchers. Firstly, the electrostatic interaction between the positively charged cationic polymer or free metal ions, and the negatively charged bacterial membrane could destroy the cell integrity. This effect might induce the increase of both hole formation and permeability of the membrane [17,31,78,88,113,114,115,116]. Secondly, photocatalytic reaction of NPs under UV and visible lights could induce the generation of reactive oxygen species (ROS) and hydrogen peroxide on the surfaces of particles [13,15,100]. This oxidative damages to proteins and DNA in the cell could provide the mainly contribution to the antimicrobial activity of NPs [15,31,76,100,117]. Finally, the detachment of free metal ions could disturb the DNA replication [13,18,97,117]. However, continuous investigations are carried out to obtain more information on and better understand the antimicrobial mechanisms of NPs.

The primary mechanism for antimicrobial activity of CS relies on the interaction between the negatively charged carboxylate (-COO-) groups on the exterior of the bacterial cell membranes and the positively charged amino (-NH_3_) groups of CS [1,4,113]. The disruption of the cell membranes is the result of this electrostatic binding process [4,114], and elevated levels of lipid peroxidation and protein leakage (Figure 5). According to Li et al. [115], amino protonation and the subsequent cationic production might be responsible for the antimicrobial activity of CS with high molecule weight since its ultra-long molecular chain was suitable for binding *E. coli* and *S. aureus*. Additionally, microbial deoxyribonucleic acid (DNA) and diffused hydrolysis product interaction could prompt protein and mRNA synthesis restriction (Figure 5) [116]. Differences in the composition, structure, thickness, and electrochemistry of the cell membrane also affect the sensitivity of different microorganisms to the inactivation of CS. More importantly, the antimicrobial activity of coating films should be mainly due to the activity of NPs incorporated into the coating films. The antimicrobial mechanism of NPs is summarized in the next section.

According to Davoodbasha et al. [17], the bactericide containing CS and AgNPs was able to destroy bacteria by interacting with the positively charged cationic polymer, and the negatively charged bacterial membrane, increasing the hole formation and the permeability of the membrane, and inducing cell death (Figure 5) [31,78,118]. As reported by Li et al. [88], the transmission electron microscopy (TEM) images showed the presence of many pits and gaps on the cell membrane when *E. coli* was exposed to AgNP. The primary hypothesis is rooted in the chemical communication of Ag^+^ ions responsible for surrounding the NPs with sulfur and nitrogen groups present in amino acids on the bacterial cells [119,120]. Furthermore, it was possible that the AgNPs disturbed the signaling pathway inside the bacterial cells resulting in the delamination of the cytoplasmic membrane and inducing the decay of cell wall [100,121,122,123,124]. The synthesized NPs caused disorganization of the bacterial cytomembrane and leakage of cytoplasmic contents [125]. Furthermore, Liu et al. [110] suggested that the embedded AgNPs could accumulate electrons, which were photogenerated in the conduction band of TiO_2_. This process produced holes in the valence of the TiO_2_ and prevented the electrons from reconnecting to the holes, inducing the increased quantum efficiency of the photochemical reaction [97,126,127,128,129]. Variations in the antimicrobial effect might be evident due to the size, shape, concentration, clarity, capping agent and surface chemistry of NPs, as well as their capacity to discharge free biocidal metal ions [130].

Yemmireddy and Hung [15] reported that the variations in the surface properties of the separate TiO_2_ nanocoatings were due to three different binders being used to create them. It is possible that the type of binders used in the TiO_2_ coating could significantly affect the photocatalytic bactericidal characteristics [13,131]. Padmavathy et al. [132] described the process as the activation of ZnO by both UV and visible light, while encouraging the generation of electron-hole pairs (e^−^/h^+^). Water molecules can be degraded into OH^−^ and H_3_O^+^ species, while dissolved oxygen molecules were converted to superoxide radical anions ·O_2_^−^ (Figure 5). These molecules would react with H_3_O^+^ to form (HO_2_·) radicals, which collided with electrons to generate hydrogen peroxide anions (HO_2_^−^). The next step was that HO_2_^−^ reacted with H_3_O^+^ to create H_2_O_2_ molecules. Doping with AgNPs could modify the visual characteristics of ZnO and further impact the antimicrobial and photocatalytic activity of Ag-ZnO composites [130,133,134]. According to Kim et al. [100], free radicals derived from the surface of AgNPs affected the membrane lipids of microorganisms, induced the oxidative damage to proteins and DNA, and caused the degradation of cell membrane [31,76,135,136,137,138].

By prompting reductive species such as superoxide anions, metal ions might also be responsible for biological damage [133]. As indicated by Manzl et al. [129], Cu ions caused further oxidative damage to bacterial cells via SOD anions and hydrogen peroxide free radicals, which were formed by TiO_2_ following exposure to sunlight [129]. Andrade et al. [18], and Marambio-Jones and Hoek [137] indicated that the disturbance of DNA replication and adenosine triphosphate production resulted from the absorption of Ag ions [97]. The proposed antimicrobial mechanism illustrates several crucial transport processes such as the uptake of phosphate and succinate, the cellular contacted oxidation, the obstructed respiratory chain, and the metal ion bonding [13].

## 4. Physical and Mechanical Properties of ECF with NPs

### 4.1. Physical Properties of ECF with NPs

#### 4.1.1. Surface Observation of ECF with NPs

The surface structure and loading capacity of NPs are critical for the applications of coating films [1,4]. Zolfi et al. [44] discovered a uniform distribution at 1% and 3% wt. loading levels of TiO_2_ NPs using SEM micrographs. While Cano et al. [14] reported sufficient distribution of the nanofiller into the polymer matrix, some TiO_2_ NPs and aggregates may appear to be adequately disseminated in the polymer matrix with elevated TiO_2_ nanoparticle content [54,66,110]. Xu et al. [5] observed that the GO-CS biopolymer acquired a rough texture following the addition of TiO_2_ NPs [8]. Moreover, Roilo et al. [139] indicated that the surface of the TEMPO-oxidized cellulose nanofiber (TO-CNF) coating was devoid of revealing pinholes and cracks, while the presence of TiO_2_ nanoparticle combinations was observed. Achachlouei et al. [50] found that SEM micrographs showed well-dispersed Na-MMT and TiO_2_ NPs through the surface of the films, especially at low concentrations. On the other hand, as reported by Valenzuela et al. [140], SEM images indicated that the superficial structures of CS and CS-quinoa protein films were devoid of pores and appeared homogeneous. However, the micropores were observed in the CS coating film as documented by Xing et al. [141], Xing et al. [94] and Xing et al. [4], which might be affected by the diverse sources and characteristics of CS, its interaction with additives, and by the various materials and preparation techniques that were employed.

SEM images generated by Singh et al. [142] indicated that conducting pathways were established between Ti substrates and the corrosive medium by the microcracks and pores, while a coating with low permeability provided a more effective barrier against the penetration or transportation of chloride ions and water molecules. The primary particles seemed to be highly agglomerated with a substantial neck formation, and their sizes displayed a visible decline with elevated Ag content [142]. These modifications in particle size and the distribution of ionic species with diverse Ag-doping levels were likely responsible for the differences in the morphology of β-Ca_3_(PO_4_)_2_ powders, as well as in the porosity of the coatings [142]. Further, SEM images of iodinated composites examined by Banerjee et al. [31], represented the petal-like structure typical of the CS matrix. TEM images signified the presence of spherical AgNP in the polymer, which denoted the generation of a stable AgNP-CS composite. Davoodbasha et al. [17] reported that TEM observation of Ch3Ag1 (3% CS solution mixed with 1 mM of AgNO_3_) exposed interlinked microporous structures with spherical AgNPs systematically dispersed within the matrix. A high probability existed that the Ag ions would have significant contact with the carbonyl, amino, and hydroxyl groups in the CS molecule for establishing structure uniformity. Usman et al. [79] studied the SEM images of PVA/AgNPs NC, and suggested that spherical AgNPs particles were entrenched in the polyvinyl alcohol (PVA) matrix without any visible accumulation. Some aggregation was evident in PVA/GO/AgNPs NC, while the visibility of the GO stacking of the four-component NC system namely PVA/GO/AgNPs/starch was possibly due to the degradation process in conjunction with AgNPs distribution. According to Andrade et al. [18], TEM micrographs of the β-CD stabilized AgNP displayed a comparatively narrow size distribution of pseudo-spherical particles. A red shift was apparent between the surface plasmon resonance (SPR) peaks of AgNPs from both types of Ag-ZnO NPs, which corresponded with broader widths for (CD) material indicating an increase in particle size dispersion. Moreover, Ortega et al. [104] suggested that AgNPs allowed for the matrix reinforcement of gelatinized starch, resulting in a more resilient material with smooth surfaces, as evidenced by SEM analysis.

In a study by Jebel and Almasi [105], SEM results showed that ultrasound (US) treatment reduced ZnO particle size, and established a hybrid nanostructure that is stable, while uniformly dispersed ZnO NPs coated the bacterial cellulose (BC) nanofibers. Shankar et al. [19] found that NC films containing Gelatin/ZnO NPs exhibited rough surfaces with evenly distributed NPs Both ZnON (ZnO NPs formed in the presence of zinc nitrate) and ZnON CMC were irregular in shape, while ZnOA (the ZnO NPs formed in the presence of zinc acetate) was cubical and ZnOA CMC was elliptical. Using SEM, Hasheminya et al. [47] determined that the CuO NPs displayed uniform distribution across the kefiran-CMC polymer matrix, while Shankar et al. [108] discovered that the SNP were dispersed evenly in the CS film. Song et al. [86] indicated that the diffused network of nano-silica in the CS matrix with the highly porous structure (TEM results), resulted from the formation of Si-O-C and hydrogen bonds [21]. SEM imaging by Lin et al. [76] revealed that the Ag/TiO_2_ NC particles were deposited onto the CS adipate layer. A possible explanation for this result is that CS acted as a reducing agent using electrostatic attraction and Ag-N coordination bonds to secure the metal ions. On the other hand, the results of Zaharia et al. [89] showed that hybrid materials modified with composite NPs (ZnO/CS and Ag:ZnO/CS) displayed the smoothest surface morphology and optimal NPs distribution.

#### 4.1.2. Thickness, Contact Angle and Tg Characterization of ECF with NPs

The thickness of edible coating films is affected by the addition of NPs. Goudarzi et al. [8] indicated that increased nano TiO_2_ content led to decreased film thickness. While contact between the nano filler and the polymer matrix might restrict water molecule distribution because of a tight network structure between TiO_2_ and starch. According to Carvalho et al. [13], a larger film thickness generated an increase in the diffraction peak intensity of the ZnO coating. Ortega et al. [104] suggested that the AgNPs content induced a minor increase in the film thickness.

Cano et al. [14] found that the contact angle increased to around 94° with the addition of 1 wt.% TiO_2_ NPs, resulting in a more hydrophobic bio-NC. Then, the integration of higher levels of TiO_2_ NPs induced a reduction in the contact angle until it reached 83°. This efficacy was the result of the TiO_2_ NPs addition that exceeded the threshold value, causing TiO_2_ to be deposited on the bio-NC exterior, diminishing its irregularity. According to the study of Goudarzi et al. [8], low TiO_2_ (1 wt.%) was responsible for an increased contact angle of the starch-based NC, while elevated levels of TiO_2_ of 3 wt.% caused the contact angle to decline. Shankar et al. [19] found that higher water contact angle (WCA) values were the result of integrating hydrophobic ZnO NPs [82,106]. Research results of Kadam et al. [64] indicated that the initial contact angle of ZP films, both with and without TiO_2_ NPs, produced diverse values that ranged from 19.6° to 25.3°, and from 17.9° to 22.8°, respectively.

Goudarzi et al. [8] found that higher TiO_2_ content raised the Tg of the film samples, which can be attributed to the formation of chemical bonds between TiO_2_ and hydroxyl groups on starch chains. Furthermore, factors such as polymer type, size, nanofiller type and uniformity of the nanofiller within the polymer matrix, play a critical role in the decline of Tm in the presence of elevated levels of TiO_2_. Oleyaei et al. [32] revealed that the inclusion of TiO_2_ NPs exerted a positive influence on both Tg and the melting point of the films [46,50,51,74].

#### 4.1.3. Transparency and Color Characterization of ECF with NPs

Cano et al. [14] determined that the effect of TiO_2_ NPs in both the UV and visible range depended on the significant reduction in the transmittance of the nano-biocomposite sheets. The lowest transmittance level was found in the presence of the highest TiO_2_ nanoparticle content, which might be attributed to the increased white coloration of the sheets with the NPs addition to the CS matrix. Goudarzi et al. [8] indicated that high TiO_2_ content prompted an increase in the L* value, ΔE, and whiteness index (WI), while the b value displayed a decline. However, Teymourpour et al. [51] reported that adding TiO_2_ NPs to the SSPS film elicited a decline in the L* value, while an increase was evident in a and b. According to Zolfi et al. [46], a rise in WI and a reduction of ΔE were apparent in the kefiran and whey protein coatings following the addition of of TiO_2_ NPs. Factors such as the difference in biopolymer type, crystallite type of the nano TiO_2_, preparation method, thickness of the film, and size of TiO_2_ might play an essential role in the color variations of the biopolymer film. Furthermore, Oleyaei et al. [74] revealed that TiO_2_ were able to restrict more than 90% of UV light while enhancing the opacity and WI of the films. Elevated levels of both TiO_2_ and MMT content were responsible for a decline in the UVB, UVA, and UVC light transmittance [139]. Cano et al. [78] analyzed the optical characteristics of the films using indicators such as internal transmittance at 450 nm (Ti) including hue (h*), clarity (L*), and chrome (C*). Moreover, a substantial decline was evident in silver-loaded films regarding the transparency (Ti), hue (h*), and luminosity (L*) values, while an increase in the color saturation (C*) was apparent. As indicated by Shankar et al. [19], the gelatin/ZnO NPs composite films displayed a lower level of transparency and presented a slight greenish yellow tint, therefore, significantly increased the total color difference value (DE) following gelatin and ZnO NPs amalgamation. Carvalho et al. [13] indicated that the ZnO thin films exhibited exceptional transparency, achieving a transmittance of approximately 80% in the visible region of the electromagnetic spectrum. On the other hand, Goudarzi et al. [8] and Hasheminya et al. [47] demonstrated that the percentage of light transmission and color specifications were usually enhanced by high nanoparticle density. According to Akbariazam et al. [52], SSPS/ZnO (4%) films exhibited 0% UV transmittance, as well as over 70% absorption of the near-infrared spectrum. Furthermore, Cano et al. [78] suggested that only transparency and color variations of the starch-PVA films were affected by the addition of AgNPs. While Arfat et al. [83] determined that FPI/FSG films exhibited a transparency reduction following the addition of BEO and ZnO NP [82].

#### 4.1.4. Thermal Stability of ECF Film with NPs

From the thermogravimetric analysis (TGA) curve, Andrade et al. [18] discovered three mass loss steps for the AgNP-β-CD. The first step referenced to the volatilization of residual water, while the second was related to the decay of β-CD molecules at 314 °C. The third step denoting extremely meager weight loss at 794 °C was ascribed to the deterioration of carbonaceous residues. As reported by Lin et al. [76], during the second stage of weight loss, it became evident that the decay of the CS/cellulose-AgNPs films was initiated at a temperature of 274.2 °C. Cano et al. [14] indicated that the TGA analysis of nano-biocomposites exhibited three degradation stages. The last peak appeared in the range of 550-800 °C and might refer to the oxidative decay of the carbonaceous residue that was produced during the second decomposition stage. Moreover, addition of TiO_2_ NPs exhibited no obvious influence on the thermal stability of the substances. Cano et al. [78] indicated the augmentation with the silver compound substantially diminished the thermal stability of the starch and PVA fractions contained in the film. Shankar et al. [19] indicated that all the films exhibited several stages of thermal degradation in the TGA curves. They also reported that the films containing ZnO NPs displayed improved thermal stability compared to the native gelatin film. Moreover, NC films exhibiting between three and four thermal decomposition steps were observed by Kadam et al. [64]. They demonstrated that the existence of degradation residue of NC specimens beyond 650 °C confirmed the presence of TiO_2_-SiO_2_ NPs (TiO_2_ as core and SiO_2_ as shell) in the samples. According to Arfat et al. [90], TGA analyses indicated that the addition of Ag-Cu NPs could enhanced the thermal stability of the NC films.

#### 4.1.5. Structural Characterization of ECF Film with NPs

X-ray diffraction (XRD) pattern analysis in the investigation by Cano et al. [14], indicated that the bio-NC contained TiO_2_ NPs in an anatase crystalline form with increasing intensity of the TiO_2_ region, and corresponded to the added TiO_2_. However, Zolfi et al. [44] established that the integration of TiO_2_ NPs did not exert any influence on the crystal type in kefiran-WPI. As reported by Xu et al. [5], with the addition of CS, the characteristic absorption peak of GO in the UV-vis spectra disappeared. However, with the addition of TiO_2_ NPs, the absorption peak appeared to be higher. Achachlouei et al. [50] found that the increase of crystallinity was prompted by the limited accumulation of TiO_2_ NPs due to the systematic arrangement of the clay nanolayers forming an intercalated structure in the NC. Moreover, the XRD test of Oleyaei et al. [74] further confirmed the completely exfoliated structure that originated in the potato starch-sodium montmorillonite (PS-MMT) NC containing 3% and 5% MMT. Cano et al. [14] indicated that the crystallinity of the biopolymer-based matrix decreased with the addition of TiO_2__._ While Salarbashi et al. [54] established that the crystalline structure of the TiO_2_ NPs remained intact in the SSPS matrix. Xu et al. [5] also indicated that in the fourier transform infrared (FTIR) spectrum, the typical C-O stretching band of the amide group shifted to a lower wave number. During the investigations of Oleyaei et al. [32], Achachlouei et al. [50], and Alizadeh-Sani et al. [66], FTIR confirmed that the same bands found between CMC and TiO_2_ NPs, also occurred between hydroxyl groups of starch and TiO_2_ nanofillers.

Considering the XRD results obtained by Lin et al. [76], it was established that the reflections seen around 34.0° and 41.0° in CS/cellulose-AgNPs composite films were assigned to the (111) and (200) planes of the AgNPs face-centered cubic, which undoubtedly confirmed the presence of AgNPs. In the FTIR spectrum, Lin et al. [76] discovered formations of amide bonds between CS/cellulose-AgNPs CS/cellulose and the primary amino groups of CS, while the carboxylic residues corresponding to the silver surface were confirmed. Mathew and Kuriakose [75] believe that the encapsulation of AgNPs could further enhance the already improved light-fastening characteristics of the chromophoric system when attached to CS. Considering the XRD analysis of PVA/AgNPs and PVA/GO/AgNPs NC, Usman et al. [79] determined that the characteristic peaks suggested the embedding of AgNPs in the related matrix. The intense OH band evident between 3550 cm^−1^ and 3000 cm^−1^ in the FTIR spectrum, was highly suggestive of H-bonding in the GO sheets at both intermolecular and intramolecular levels. A reduction in the intensity of peaks located at 1722 cm^−1^, 1421 cm^−1^, and 1370 cm^−1^, indicated the disengagement of these vibrations due to AgNPs contact with the OH groups of PVA [79]. In a PVA-GO-Ag-starch film, the movement that occurred on the OH stretching vibration peak, as well as the CO H stretching peak to a lower wave number signified substantial molecular interactions [79]. Moreover, Andrade et al. [18] noted that the plasmon peak was visible around 405 nm, which could be ascribed to the development of pseudo-spherical AgNPs and corresponded to the surface resonance of these NPs. Both Abou-Okeil et al. [143] and Andrade et al. [18] indicated the interaction of chemisorption between the OH group in β-CD and the AgNP surface, which might be responsible for the loss of β-CD crystallinity.

According to Shankar et al. [19], the XRD patterns of the NC films containing various types of ZnO NPs displayed typical diffraction peaks, which provided confirmation that the crystalline ZnO NPs presented in the NC films. Furthermore, as reported by Arfat et al. [90], XRD emphasized the presence of a change in the crystallinity of FSG films following the addition of NPs [19,105]. During a microstructural analysis by Arfat et al. [83], it was found that the presence of ZnO NP inhibited bilayer formation of a film containing 100% BEO. Shankar et al. [19] utilized the FTIR spectrum to reveal contact between ZnO NPs and N-H groups of gelatin. According to Malini et al. [81], the fluorescence emission intensity of nano ZnO was significantly higher than that of the NC, as evidenced by a blue shift (from 360 nm to 335 nm) in the UV-visible spectrum of NC.

Examining XRD patterns, Carvalho et al. [13] discovered that several major peaks of ZnO were evident in the ZnO(Ag)-1 and ZnO(Ag)-2 samples, which denoted low crystalline preferential growth. From the XRD patterns and the FTIR spectra conducted by Singh et al. [142], it was found that the amide bands shifted to a lower wavelength. These findings in conjunction with the change in the intensity of Infrared (IR) transmission at the CH_2_, CH_3_, amine, and carbonyl bands were compared with those of CS and found to be suggestive of contact between CS, β-Ca_3_(PO_4_)_2_ and silver ions. Hasheminya et al. [47] indicated that hydrogen bond formation in the polymer matrix with CuO NPs and the crystalline structure in the CuO NPs polymer were revealed by FTIR spectroscopy and XRD analysis, respectively. Shankar et al. [19] utilized FTIR analysis to reference the intensity increase in the amide-A region, which suggested that hydrogen bonding was employed to initiate contact between N-H groups of gelatin protein chains and ZnO NPs. According to Lungu et al. [111], a red shift with 1 nm was evident with reference to the Ag-ZnO NPs (mechanical deposition, MD) specimen, while a blue shift of 10 nm appeared to signify the Ag-ZnO NPs (CD) sample. These shifts were closely related to the Ag-ZnO NPs size, as well as contact with the electron-hole exchange on the NPs surface. AgNPs loading onto ZnO NPs surface substantially elevated the absorption levels within the visible light spectrum. The presence of (COO-), (-OH) and (-CH_2_) groups confirmed CMC capping of the synthesized Ag-ZnO NPs samples.

### 4.2. Mechanical Properties of ECF with NPs

The operational qualities of ECF with NPs were essential for the application of CS-based coatings, to allow for analysis regarding the prevention of moisture loss in fresh produce. Cano et al. [14] reported that CS matrices fortified by adding TiO_2_ NPs significantly enhanced the tensile strength (TS) and the Young’s modulus (YM) impacting elongation at break (EB). Goudarzi et al. [8] also indicated that water-vapor permeability (WVP) of starch/TiO_2_ NC exhibited a decline in the presence of high TiO_2_ content of 0% to 1% and 3%, while an increase was evident when the TiO_2_ content reached 5%. Moreover, TS of the starch/TiO_2_ NC functioned independently from the TiO_2_ content, while high TiO_2_ content induced a rise in the EB and tensile energy to break (TEB). The addition of TiO_2_ to the films prompted a minor rise in TS, as well as a reduction in EB. Teymourpour et al. [51] determined that while moisture content, WVP, and oxygen permeability were reduced by addition of TiO_2_ to the SSPS matrix, the process successfully enhanced the mechanical properties. The addition of REO and TiO_2_ substantially increased the water resistance of WPI/CNFs composite films according to Alizadeh-Sani et al. [45]. Additionally, the amalgamation of WPI/CNFs 7.5% films with both REO and TiO_2_, displayed lower EB, and higher TS and YM. Research of Zolfi et al. [44] suggested that adding TiO_2_ NPs to kefiran-WPI films induced a substantial decline of TS and YM, and increased its EB. Goudarzi et al. [8] established that although high TiO_2_ content prompted a reduction in the TS and YM of the film samples, it induced a rise in both the EB and TEB. Moreover, Vejdan et al. [144] determined that the WVP of the gelatin/agar-TiO_2_ bilayers was reduced by the addition of TiO_2_, while a rise in moisture content was evident with high nano-TiO_2_ content. Even though the TS in the bilayer films displayed an increase in conjunction with high nano-TiO_2_ content, a further increase prompted a reduction of the TS. According to Oleyaei et al. [32], the addition of MMT and TiO_2_ improved the EB and TS of the coatings, while the WVP displayed a decline [78]. Achachlouei et al. [50] determined that the nanoclays enhanced the resilience of the NC to the YM and tensile stress of the films to the detriment of EB. A possible explanation for this contrasting behavior might be an oversaturation of the active polymer network points for silver adsorption. Usman et al. [79] showed that the TS and YM of PVA varied significantly with the addition of GO, AgNPs and starch.

Arfat et al. [83] characterized the FPI/FSG films containing basil leaf essential oil and ZnO. By adding ZnO NP, the TS displayed higher levels, while a reduction was evident in the EAB. The lowest WVP was achieved in the film containing 100% BEO and 3% ZnO NP. Arfat et al. [90] determined that an increase in TS and a decrease in EB were characteristic of NC films containing 2% (*w*/*w*) NPs. Additionally, Shankar et al. [19] established that the TS of NC films containing CMC capped ZnO NPs appeared to be slightly higher than those that were uncapped. Furthermore, by adding ZnO NPs, the EB and WVP of gelatin films displayed a substantial increase. According to Meindrawana et al. [43], the elongation limits of the carrageenan/ZnO (CZ) NPs NC film exhibited no significant increase compared to carrageenan film. Shankar et al. [19] demonstrated that the moisture content, WVP, and EB of ZnO NPs modified films were higher than those in the control gelatin film. Akbariazam et al. [52] revealed that adding 4% ZnO into the SSPS matrix enhanced the EB and heat seal strength of the films. Jebel and Almasi [105] established that ZnO NPs improved the mechanical characteristics of BC films, while reduced WVP and moisture absorption. According to Nafchi et al. [82], the introduction of low concentrations of the nanorod-rich ZnO (ZnO-N, the ZnO NPs formed in the presence of zinc nitrate) to starch solutions significantly decreased the WVP of the films. Hasheminya et al. [47] indicated that an increase in the CuO NPs concentration improved the TS and decreased the WVP and EB of the kefiran-CMC coating film. Shankar et al. [108] showed that the SNP incorporation enhanced the mechanical strength and WVP of the CS film. Kadam et al. [64] suggested that the mechanical properties of ZP films improved with the addition of TiO_2_-SiO_2_ NPs (1.5%, *w*/*w*). However, NPs did not display any significant effect on the WVP.

### 4.3. Gas Modification Property of ECF with NPs

**Figure 6:**
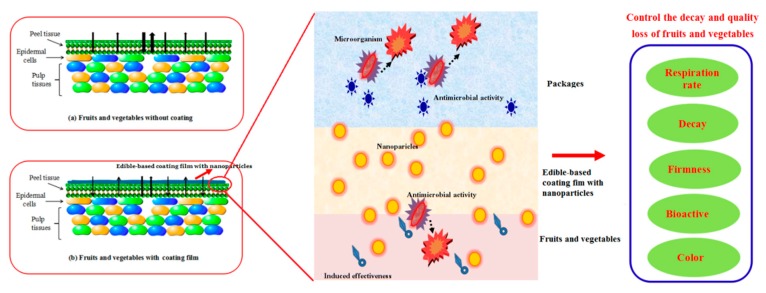
Preservation mechanism of ECF (such as CS coating film) with NPs on the quality of pulps.

The preservation mechanism including gas modification and induction defense properties of ECF with NPs were demonstrated in Figure 6. As indicated in Figure 6, the solution of ECF with NPs can form a thin coating film on the surfaces of fresh products after being treated and dried. Then, the matrix carriers such as CS-based coatings with semipermeable properties could retard the respiration rate of fruits by modifying the concentrations of O_2_, CO_2_, and ethylene in the packages. This gas modification might inhibit the loss of water and nutrients in the products [1,4,68,94]. On the other hand, the NPs in coating materials on the produce surface might exhibit excellent antimicrobial activities, especially induced by UV light, against pathogens in the packages and grown on the surface of fresh products. These antimicrobial activities could provide the significant contributions for controlling the decay and quality loss of produce. Moreover, some coating materials such as CS also might provide the antimicrobial activities against bacteria and fungi during the storage of fruits and vegetables [4]. More importantly, as shown in Figure 6, the ECF such as CS with NPs could possibly reduce the free radicals and activate the defense-related enzymes in the fruits cells, and then induce the defense activity of fruits and vegetable during the storage period [4,86]. This combined effectiveness would keep the firmness, bioactive and inside color and then maintain a good quality and a suitable shelf life of treated fresh products (Figure 6).

The respiration rate of fruit pulps in the packaging system is related to the speed with which nutrients are consumed. Therefore, the decline in nutritious value occurs more slowly when the respiration rate is low (Figure 6). Roilo et al. [139] demonstrated that PLA as a transparent biopolymer exhibited the capacity for high gas permeability, which might depend on the integrity of the particular coating film, the number of micro-pores, the thickness of the coating, as well as the interaction between the coating polymer and the additives. Sun et al. [85] indicated that the SiOx dosage affected the micro-morphology and microstructure of CS coatings. Therefore, high nano SiOx content could induce significant aggregation and a coarse cross-section. Moreover, substantial hydrogen bonds might form between nano SiOx and CS molecules, while the reorganization of the CS structures could occur. As indicated by Chi et al. [57], the permeability of the films was affected by the addition of NPs, which offered the possibility to further slow the respiration rate and reduce Vc consumption in pulps (Figure 6). The reason for this might be that fruit ripening was inhibited by the modification induced by NC films of the internal atmosphere such as ethylene, CO_2_, and O_2_. Additionally, Roilo et al. [139] established that the incorporation of TiO_2_ NPs decreased the film permeability by elevating the irregularity of the migration path. The sub-nanometer sizes of the empty spaces between the cellulose nanofibrils were closely related to the established barrier characteristics, as well as to the preferred transportation of small-sized penetrants [145,146]. Incorporating of TiO_2_ NPs had little change in the penetrant transport mechanism, while possible interface modifications and imperfections in the void structure influenced the penetrant diffusivity to a minimal extent. As a buffer against water vapor and critical gases O_2_, TiO_2_-loaded TO-CNF coatings offered a promising technical solution [139,147]. Meindrawana et al. [43] determined that adding ZnO NPs to carrageenan coatings inhibited the gas exchange on fruit surfaces [148]. In accordance with the water vapor transmission rate (WVTR) results, a high concentration of ZnO NPs was evident in the coating material creating a high moisture barrier in the film. The accumulation of ZnO NPs during the formation of the coating material possibly affected the capacity of the coating material to act as a barrier. ZnO NPs contributed in restricting the exchange of carbon dioxide and oxygen during the respiration process. Li et al. [11] provided confirmation by establishing that the oxygen transmission rate of normal packaging exceeded that of ZnO NPs-based packaging. ZnO coatings with NPs exhibited selective gas permeability and reduced the exchange of O_2_ and CO_2_ in fruit. Moreover, the ZnO NPs filler in the coating solution presented the ability to improve the barrier properties of the polymers by increasing pathway irregularity [149].

## 5. Applications of ECF with NPs on the Quality of Fruits and Vegetables

### 5.1. Induction Defense Properties of ECF with NPs

During the entire storage period, reactive oxygen with a stronger oxidizing ability caused severe damage to the cell membranes of fresh produce. Defense enzymes such as peroxidase (POD), superoxide dismutase (SOD) and catalase (CAT) were able to remove the factors leading to degradation [1,4,93]. Furthermore, Hong et al. [150] reported that lipid peroxidation, an oxidation process that produces unsaturated fatty acids via free radical action, was harmful to pulp cells [21]. The ECF with NPs could possibly reduce the free radicals and activate the defense-related enzymes in fruits, which could be attributed to the individual or combined action of coating carriers and NPs (Figure 6) [4,93]. Moreover, CS treatment induced a significant increase in the activities of CAT and SOD, and inhibited the production of superoxide radicals, which possibly delayed content modifications in MDA and the cell membrane permeability of fruit [94,150]. This efficacy was dependent on the concentration of CS used. The molecular weight of CS might attribute to the antioxidative influence of active compounds in the coating film and the transfer of free electrons from the peroxide to the CS electron sink [1,4,151]. Singh et al. [152] indicated that no significant loss was evident in the moisture content of vegetables from NPs-impregnated packets, but the loss in those without the addition of AgNPs impregnation was considerable. The control of moisture content and the nutritional profile in pockets containing NPs might be related to the cell permeability of vegetables.

### 5.2. Metal-Ion Release and Migration Properties of NPs from ECF

Free metal-ions released from the surface of carriers played an antimicrobial role in eradicating microorganisms on the exterior of agricultural products [97]. An investigation by Cano et al. [78] indicated that the simulants A (ethanol 10% (*v*/*v*)), C (ethanol 20%(*v*/*v*)), and D1 (ethanol 50% (*v*/*v*)) displayed similar release profiles of silver, while simulants B (acetic acid, 3% (*w*/*v*), with low pH) and D2 (oleic acid as a vegetal oil, non-polar medium) behaved differently. The release of silver was encouraged by the water absorption of the film in aqueous systems. Most of the release occurred within the first 60 min, while 100% of the silver was released following 60 min of immersion in the acidic medium (simulant B). Furthermore, Liu et al. [110] examined the silver ions released from the Ag/TiO_2_ composite films, and noted a rapid increase in the released quantities during the first five days, followed by a steady decline in the release rate. Additionally, Wei et al. [97] established that Cu ion concentrations were released from the plastic coatings at 1 wt.%, while the release rate gradually declined after 5 h. According to Salarbashi et al. [54], no TiO_2_ was detected by inductively coupled plasma optical emission spectroscopy (ICP-OES) in bread samples covered with SSPS/TiO_2_ film and kept in storage for six months. A minuscule amount of TiO_2_ released from the NC films was observed in water. More importantly, long-term exposure of cells to uncoated NPs elicited that the presence of TiO_2_ NPs were in the plasma membrane of the epithelial cell line. Liu et al. [110] determined that a slower release behavior of silver ions occurred in mesoporous Ag/TiO_2_ films in comparison with bulk matrixes.

The release of NPs or metal-ions into a system is affected by various factors. These factors include, but are not limited to the microstructure of the coating carrier, the particle and ion diffusion to the medium via the polymer, medium migration to the polymer matrix and its expansion, as well as the polymer solubility in the medium phase [78]. Moreover, the polymer chains might be rearranged by increasing the immersion and storage times. This process could increase the intermolecular interactions, decrease the mobility of polymer chains, and control the dissolved and released NPs to some extent [54,78]. Furthermore, as indicated by Cano et al. [78], different release behaviors in the non-polar simulant might be induced by the limited diffusion of oleic acid in the polar matrix by maintaining a closed network structure for the carrier matrix. However, by increasing the ethanol concentrations in the stimulant, the polarity of medium could be reduced and the hydration process of the polymer network could be controlled, and possibly silver diffusion was reduced. On the other hand, the film compositions and initial NPs concentrations also affect the NPs release [99,103]. This efficacy might further modify the differences in antimicrobial activity due to variations on the surfaces of free ions or NPs that are thought responsible for inhibiting the microorganisms.

### 5.3. Effect of ECF with NPs on the Quality of Fruits and Vegetables

Coating film could be used as carriers for metal NPs as antimicrobial agents, as well as protective barriers, which might reduce the respiration rates, control the decay and color changes, maintain the storage quality, and prolong the shelf life of fresh produce [4,95].

Xu et al. [5] compared uncoated strawberries and mangos with samples exposed to NC, and stored at room temperature for 7 d. They found that the coated samples exhibited a lower weight loss and maintained a better appearance, while polyphenol oxidase (PPO) activity was inhibited by the NC coating containing GO and CS loaded TiO_2_. SOD activity in fruits coated with three different types of NC films exhibited higher values than the untreated samples and consequently, indicating a significant potential in the food preservation industry.

Chi et al. [57] employed mangoes to determine the effects of PLA/BEO/nano-TiO_2_ film and PLA/BEO/nano-TiO_2_ + nano-Ag film on their quality. Comparison results with PLA and PLA/BEO films indicated that PLA NC films successfully restricted adverse changes in total acidity, vitamin C content, and color, while firmness retention in the mangoes was enhanced. Moreover, PLA NC films inhibited the growth of microorganisms on the mango pericarp. The quality of mangoes exposed to the PLA NC films remained acceptable following prolonged storage periods, and results showed that the shelf life of these fruits was extended up to 15 d.

Meindrawana et al. [43] established that on day 13 of storage, the decline in the total acidity of mangoes exposed to CZ 0.5 and CZ 1 NP coating treatments was significantly lower than that in the control samples. The CO_2_ production peak occurred after 7 d of storage, where the mangoes in the control sample produced the highest level of CO_2_. Until day 33, minimal degradation was evident in the mangoes with CZ 0.5 coatings compared to samples without coatings. Compared with carrageenan coatings, adding ZnO NPs promoted weight loss in the fruit.

According to Costa et al. [153], samples of control (fresh fruit salad without silver-montmorillonite (Ag-MMT)), 10-NP (fruit salad with 10 mg of Ag-MMT), and 15-NP (fruit salad with 15 mg of Ag-MMT) exhibited signs of decay before the 20-NP (fruit salad with 20 mg of Ag-MMT) specimen, which were consistent with the microbiological results. The shelf life of each tested sample was calculated based on its sensorial and microbial qualities. This experiment found that 20 mg Ag-MMT NPs could maintain the sensorial characteristics of fresh fruit salad during prolonged storage time.

Yu et al. [20] indicated that jujube coated with CS + nano-SiO_2_ displayed the lowest red index value, as well as the lowest respiration rate among all the treatments. Moreover, this composite coating maintained high SOD, POD, CAT levels, while reducing phenylalanine ammonia-lyase (PAL) activity, delaying MDA elevation, and preserving the total fruit flavonoids.

Luo et al. [154] obtained similar results by coating freshly cut-asparagus with a combination of CS and SiOx. Results indicated that the respiration rate and ethylene production rate decreased while the activities of PAL, PPO, and POD increased rapidly. Moreover, the accumulation of total phenolic compounds was enhanced, while the same coating application displayed potential effectiveness for the quality retention of fresh-cut bamboo shoots.

Meng et al. [148] determined that combining of 1.2 g L^−1^ nano-ZnO coating with ultrasound treatment at 40KHz prompted a reduced rate of ethylene and CO_2_ production, water loss, and texture degradation in fresh-cut kiwi, compared to other treatments at the end of storage. Therefore, this combination represented a promising approach for maintaining the quality of fresh-cut kiwi.

Song et al. [86] established that the use of CS/nano-silica coating significantly delayed the browning and weight loss of loquat fruit, and maintained the titratable acid (TA) levels in the fruits. Moreover, PAL, PPO, and lipoxidase activity were effectively inhibited in the coated fruits. This coating might sufficiently extend the shelf life of loquat fruit with improving its chilling tolerance in the process.

Ortiz-Duarte et al. [155] demonstrated that compared to uncoated samples, Ag-CS NC coatings obtained from red claw crayfish successfully maintained a lower respiration rate, controlled the loss of firmness, retained high total vitamin C content, while reduced translucency and promoted good sensory characteristics during storage. Therefore, this coating displayed potential as an application in the fresh produce industry to extend the shelf life of these products.

Shah et al. [156] had investigated the aromatic profile and organoleptic qualities of kinnow exposed to an AgNPs coating consisting of CMC and guar gum. The sensory qualities of both coated and uncoated fruit stored at 10 °C declined during the storage period. Significant losses of linalool, limonene, and γ-terpinene content were prevented by the application of gum-Ag coatings and storage at 4 °C. Kinnow coated with the coating kept the sensory quality and fruit aroma for 120 d at a storage temperature of 4 °C.

Shi et al. [21] indicated that fruits treated with the CS-silica NC exhibited significantly less lesion formation and browning than the control samples. This coating succeeded in preventing weight loss, while restricted PPO and POD activity in longan during storage. Moreover, by safeguarding the membrane structure against peroxidation, this coating substantially delayed the formation of MDA, obstructed the production of TA and restricted Vc loss in fruits. These effectiveness might lead to delay the senescence and decomposition of fruit.

Singh et al. [152] investigated the biochemical properties of vegetables following exposure to cellulosic packaging. The retention of freshness during storage was obtained by measuring the moisture content of the vegetables. Vegetables stored in packaging containing AgNPs were subjected to ethanol extracts and found to be rich in phenols, proteins, total antioxidants, and flavonoid content. However, being exposed to packaging containing AgNPs displayed no substantial impact on the nutritional profile of the vegetables.

The research works discussed in this section indicated that ECF enriched with different NPs could delay the respiration rate, decrease weight loss, and retain the nutrients and sensory quality of fruits and vegetables.

## 6. Future Trends of ECF with NPs

Many materials have been developed to prepare coating films for the storage of fruits and vegetables recently. The evaluated works on the properties of coating films prepared by various edible materials are important for their further application in food storage. Therefore, the main benefits and drawbacks of ECF with different materials, especially complex materials should be demonstrated, which might provide some important information for further using these NPs in the coating films.

In recent years, nanosized particles as antimicrobial agents incorporated into edible coatings were the subject of many studies. However, additional research involving the interactions between nanosized particles and coating materials are necessary. Moreover, further works about the effect of these NP addition on the properties of coating materials properties should be understood. These investigation results might provide insights regarding further improvements to their physical and antimicrobial properties for practical applications.

The antimicrobial activity of edible coatings containing nanosized particles is a critical factor for its evaluation and application, while its antibacterial activity and mechanism are popular research topics. Although the antifungal activity of ECF incorporated with NPs has also been investigated, few articles about its antifungal mechanism are published. Therefore, the antifungal mechanism, especially under the induction of UV or visible light requires further investigation.

The toxicity of edible coatings containing nanosized metal particles should be examined extensively since it possesses the potential to induce allergic reactions, especially in freshly cut products due to the release and migration of metal ions. On the other hand, the main quality factors of fruits and vegetables that might be improved or reduced by the application of various ECF with NPs should also be indicated. Therefore, further research on these subjects are essential.

It is vital for further research to focus on enhancing the consistency of composite coating properties, and to investigate its effects on the storage quality of fruits and vegetables to establish the standards for its preparation and application [4]. Further studies involving cost-effective ways to prepare and apply ECF are necessary.

Following application, the NC can attach uniformly to the pulp pericarps, achieving stability even when cracks are present. Consequently, bacteria are possibly obstructed from gaining entry to these spaces. Further research should investigate the toxicity and safety of these coatings, the possibility of NPs migration from the coating to fruit, as well as the risks involved in human consumption [87].

## 7. Conclusions

To effectively maintain the storage quality and prolong the shelf life of fresh products, the novel ECF containing different NPs was developed increasingly as a convenient technique. In this paper, the properties of main edible-based coating materials were first introduced. Then, the ECF with NPs such as TiO_2_, Ag, and ZnO developed by other researchers was reviewed. The antimicrobial activity of ECF with NPs against the tested microorganism were observed by researchers. Several antimicrobial mechanisms including the electrostatic interaction between the cationic polymer or free metal ions and the charged cell membrane, the photocatalytic reaction of NPs and the detachment of free metal ion are summarized and introduced. Thereafter, a discussion was included on the surface structure, and mechanical properties of ECF with NPs, which are crucial for its applications of fruits and vegetables during storage at refrigerated or room temperatures. The properties including gas modification, induction defense and ion release of ECF with NPs were also discussed. On the other hand, results from additional works indicated that the applications of ECF with different NPs might provide a suitable and effective way to prevent the quality loss in postharvest fruits and vegetables. More importantly, additional research involving the interactions between nanosized particles and coating materials, and the antifungal mechanism inducted by UV or visible light require for further investigation. The works on the prepared and used standards and the safety of these coatings also need to be carried out in the future.

## Figures and Tables

**Table 1:** Summary of different NPs and main edible-based materials used in coating films.

NPs		Main Edible Materials	Reference
TiO_2_		Shellac	Yemmireddy and Hung [15]
		CS, WPI	Cano et al. [14], Xu et al. [5], Zhang et al. [73]
		Potato starch	Oleyaei et al. [74]
		SSPS	Teymourpour et al. [51]
		WPI/CNFs	Zolfi et al. [44], Alizadeh-Sani et al. [66]
		CMC	Achachlouei et al. [50]
Ag		CS	Davoodbasha et al. [17], Mathew and Kuriakose [75], Lin et al. [76]; Kumar-Krishnan et al. [77]
		Starch, PVA	Cano et al. [78], Usman et al. [79]
		β-CD	Andrade et al. [18]
		Agar, banana powder	Orsuwan et al. [80]
ZnO		CS	Malini et al. [81]
		Sago starch	Nafchi et al. [82]
		FPI, FSG	Arfat et al. [83]
		MC	Espitia et al. [84]
		SSPS	Akbariazam et al. [52]
Others	Silicon dioxide	CS	Yu et al. [20], Sun et al. [85]
	Silica	CS	Shi et al. [21], Song et al. [86]
	CuO	Kefiran, CMC	Hasheminya et al. [47]
	SNP	CS	Shankar et al. [19]
Mixed NPs	Ag-TiO_2_	CS	Lin et al. [87]
	TiO_2_-Ag	PLA	Chi et al. [57]
	Ag-Cu	FSG	Arfat et al. [90]
	TiO_2_-SiO_2_	ZP	Kadam et al. [64]

**Table 2:** Summary of the tested microorganisms inhibited by various ECF with NPs.

Main Edible Materials and NPs	Inhibited Microorganisms	Reference
WPI/CNFs, TiO_2_	*L. monocytogenes*, *S. aureus*, *E. coli O*_157_:*H*_7_, *P. fluorescens*, *S. enteritidis*	Alizadeh-Sani et al. [66]
Polylactide, TiO_2_	*L. monocytogenes*, *S. Typhimurium*, Shiga toxin producing *E. coli*	Huang et al. [99]
SSPS, TiO_2_	*E. coli*, *S. aureus*	Teymourpour et al. [51]
SSPS, TiO_2_	*S. aureus*	Salarbashi et al. [54]
CS, Ag	*E. coli*, *S. aureus*	Kumar-Krishnan et al. [77]
CS, Ag	*S. choleraesuis*, *S. aureus*, *B. subtilis*, *E. coli*	Jia et al. [101]
Poly(ε-caprolactone), curcumin, grape leaf extract, Ag	*E. coli*, *S. aureus*, *S. enterica*, *P. aeruginosa*, *B. subtilis*, *C. albicans*, *A. flavus*	El-Sherbiny et al. [102]
CS, Ag	*E. coli*, *P. aeruginosa*, *V. parahaemolyticus*, *V. vulnificus*, *S. aureus*, *B. cereus*, *C. albicans*, *A. parasiticus*	Davoodbasha et al. [17]
CS, Ag	*S. aureus*, *E. coli*, *A. terreus*, *A. flavus*	Mathew and Kuriakose [75]
CS, Ag	*E. coli*, *S. aureus*,	Lin et al. [76]
Starch, PVA, Ag	*A. niger*, *P. expansum*, *L. innocua*, *E. coli*	Cano et al. [78]
CS, ZnO	*B. substili*, *K. planticola*	Malini et al. [81]
CS, ZnO	*S. enterica*, *E. coli*, *S. aureus*	Al-Naamani et al. [106]
FPI, FSG, ZnO, BEO	*L. monocytogenes*, *P. aeruginosa*	Arfat et al. [83]
Kefiran, CMC, CuO	*S. aureus*, *E. coli*	Hasheminya et al. [47]
CS, SNP	*E. coli*, *L. monocytogenes*	Shankar et al. [108]
CS, Ag-TiO_2_	*E. coli*	Lin et al. [87]
CS, Ag, ZnO	*E. coli*, *B. subtilis*, *Aspergillus*, *Rhizopus*, *Penicillium*, *S. aureus*, *yeast*	Li et al. [88]
